# Nitric oxide protects the heart from ischemia-induced apoptosis and mitochondrial damage via protein kinase G mediated blockage of permeability transition and cytochrome *c *release

**DOI:** 10.1186/1423-0127-16-70

**Published:** 2009-08-11

**Authors:** Vilmante Borutaite, Ramune Morkuniene, Odeta Arandarcikaite, Aiste Jekabsone, Jurgita Barauskaite, Guy C Brown

**Affiliations:** 1 Institute for Biomedical Research, Kaunas University of Medicine, Kaunas, Lithuania; 2 Department of Biochemistry, Kaunas University of Medicine, Kaunas, Lithuania; 3 Department of Biochemistry, University of Cambridge, Cambridge, UK

## Abstract

**Background:**

Heart ischemia can rapidly induce apoptosis and mitochondrial dysfunction via mitochondrial permeability transition-induced cytochrome *c *release. We tested whether nitric oxide (NO) can block this damage in isolated rat heart, and, if so, by what mechanisms.

**Methods:**

Hearts were perfused with 50 μM DETA/NO (NO donor), then subjected to 30 min stop-flow ischemia or ischemia/reperfusion. Isolated heart mitochondria were used to measure the rate of mitochondrial oxygen consumption and membrane potential using oxygen and tetraphenylphosphonium-selective electrodes. Mitochondrial and cytosolic cytochrome *c *levels were measured spectrophotometrically and by ELISA. The calcium retention capacity of isolated mitochondria was measured using the fluorescent dye Calcium Green-5N. Apoptosis and necrosis were evaluated by measuring the activity of caspase-3 in cytosolic extracts and the activity of lactate dehydrogenase in perfusate, respectively.

**Results:**

30 min ischemia caused release of mitochondrial cytochrome *c *to the cytoplasm, inhibition of the mitochondrial respiratory chain, and stimulation of mitochondrial proton permeability. 3 min perfusion with 50 μM DETA/NO of hearts prior to ischemia decreased this mitochondrial damage. The DETA/NO-induced blockage of mitochondrial cytochrome *c *release was reversed by a protein kinase G (PKG) inhibitor KT5823, or soluble guanylate cyclase inhibitor ODQ or protein kinase C inhibitors (Ro 32-0432 and Ro 31-8220). Ischemia also stimulated caspase-3-like activity, and this was substantially reduced by pre-perfusion with DETA/NO. Reperfusion after 30 min of ischemia caused no further caspase activation, but was accompanied by necrosis, which was completely prevented by DETA/NO, and this protection was blocked by the PKG inhibitor. Incubation of isolated heart mitochondria with activated PKG blocked calcium-induced mitochondrial permeability transition and cytochrome *c *release. Perfusion of non-ischemic heart with DETA/NO also made the subsequently isolated mitochondria resistant to calcium-induced permeabilisation, and this protection was blocked by the PKG inhibitor.

**Conclusion:**

The results indicate that NO rapidly protects the ischemic heart from apoptosis and mitochondrial dysfunction via PKG-mediated blockage of mitochondrial permeability transition and cytochrome *c *release.

## Background

Endogenous or exogenous nitric oxide (NO) can protect the heart from ischemia plus reperfusion-induced damage, but the mechanisms of this protection are not entirely clear [1,2]. Suggested mechanisms include: improving coronary vascular perfusion, decreasing monocyte infiltration, improving contractile function, opening of mitochondrial K^+^_ATP _channels, inhibition of mitochondrial respiration, inhibition of mitochondrial permeability transition or inhibition of apoptosis [2]. The mechanism or mechanisms are important because NO can also damage the heart [3-5], and potently lowers blood pressure making it impractical to use clinically.

Ischemic preconditioning (a short, non-damaging, period of ischemia followed by reperfusion) is known to strongly protect the heart against a subsequent, longer period of ischemia/reperfusion. NO has been implicated in both triggering the protection during preconditioning and mediating the protection during the subsequent ischemia [1,6,7]. Most studies have indicated that the protective effect of NO is mediated by stimulation of soluble guanylate cyclase to produce cGMP, which then activates protein kinase G (PKG) [8], although other studies have suggested that protection is mediated by NO inhibition of mitochondrial respiration [9,10], or S-nitrosylation of proteins such as COX-2 [5]. PKG is thought to protect via either vasodilation [11], contractility [12], calcium transport [13], or activation of a mitochondrial K^+^_ATP _channel [14].

In contrast to reperfusion-induced necrosis, relatively little is known about ischemia-induced apoptosis: the main subject of this study. It is important to distinguish between the effects of ischemia and the effects of reperfusion on the heart. Necrosis does not occur during ischemia, but rather during the subsequent reperfusion, and this has been attributed to either production of reactive oxygen and nitrogen species, pH elevation or calcium uptake as a result of the return of oxygen, and all of these can trigger mitochondrial permeability transition. Permeability transition is a large increase in the permeability of the inner mitochondrial membrane caused by reversible pore formation, induced by high calcium and/or oxidants, and inhibited by ATP, acid pH and cyclosporine A. However, we have previously shown that heart ischemia (in the absence of reperfusion) results in rapid release of cytochrome *c *from mitochondria into the cytosol, causing both activation of caspases (and subsequent nuclear apoptosis) and inhibition of the mitochondrial respiratory chain (which might contribute to necrosis at reperfusion) [15]. And we found that all of these ischemia-induced events are blocked by inhibiting the mitochondrial permeability transition pore [15], suggesting that ischemia induces permeability transition, which triggers cytochrome *c *release. The role of apoptosis in ischemic damage to the heart is still unclear, but inhibition of apoptosis in a variety of animal models has been shown to protect the heart from ischemic/reperfusion damage [16,17], indicating that apoptosis can contribute to this heart pathology.

It is not known whether NO can protect the heart via acutely inhibiting ischemia-induced apoptosis, therefore we sought to determine what effects acute addition of an NO donor might have on ischemia-induced mitochondrial dysfunction, cytochrome *c *release and caspase activation, and by what mechanism.

## Methods

The procedures used in this study were in compliance with the *European Convention for the protection of vertebrate animals used for experimental and other purpose*.

Hearts from 2–4 months old male Wistar rats were used in all experiments. Animals were divided into three main groups: control, ischemia and ischemia-reperfusion groups. The ischemic and ischemic-reperfusion groups were further divided into several subgroups in which hearts were pre-treated with DETA/NO in the presence/absence of various inhibibitors. All hearts were perfused on a Langendorff perfusion system with Krebs-Henseleit solution (11 mM glucose, 118 mM NaCl, 25 mM NaHCO_3_, 4.8 mM KCl, 1.2 mM KH_2_PO_4_, 1.2 mM CaCl_2_, 1.7 mM MgSO_4 _and 0.7 mM Na pyruvate, saturated with 95% O_2_-5% CO_2_, pH 7.4 at 37°C) at a pressure of 80 cm H_2_O. After a 15-minute equilibration period 50 μM DETA/NO (Alexis) was added to the perfusion buffer and hearts were perfused for another 3 min with buffer containing DETA/NO. Control hearts were perfused for 20 min (15 min equilibration plus additional 3–5 min) with buffer only. After the equilibration period (and perfusion with DETA/NO where appropriate) hearts were subjected to 30 minutes stop-flow global ischemia. In reperfusion experiments, hearts were subjected to 30 minutes stop-flow global ischemia followed by 30 min reperfusion. In some experiments where inhibitors were used (1 μM KT 5823, the protein kinase G inhibitor from Calbiochem, 25 μM 1H-[1,2,4]oxadiazolo [4,3-a]quinoxalin-1-one (ODQ), the guanylyl cyclase inhibitor from Sigma, or 100 μM 5-hydroxy decanoate (5-HD), the mitochondrial K^+^_ATP _channel blocker from Sigma, 0.5 μM Ro 32-0432 and 1 μM Ro 31-8220, the protein kinase C inhibitors from Calbiochem and Sigma, respectively) they were added to the perfusion media 5–15 min prior to perfusion with DETA/NO. Data obtained in each group were compared to control group and ischemic or ischemic/reperfusion group, respectively.

Steady-state level of NO released from 50 μM DETA/NO was measured in 1 ml of oxygenated perfusion buffer at 37°C using NO sensitive electrode and was found to be 281 ± 55 nM.

KCl-based buffers were used for mitochondrial isolation in order to get higher yields of mitochondria. Hearts were cut into small pieces and homogenized with a teflon-glass homogenizer in the isolation buffer (10 ml/g of tissue) containing 180 mM KCl, 20 mM Tris HCl, 1 mM EGTA, pH 7.3 at 4°C temperature. Cytosolic and mitochondrial fractions were separated by differential centrifugation (5 min × 750 g, 10 min × 6800 g). The post-mitochondrial supernatant was additionally centrifuged for 30 min at 10000 g and the resulting supernatant (S_10_) was used for determination of cytochrome *c *content in cytosol. Total cytosolic and mitochondrial protein was measured by a Biuret method.

Mitochondrial respiration rate and membrane potential were measured with a Clarke-type oxygen electrode and a tetraphenylphosphonium-selective electrode as described in [18]. The composition of mitochondrial incubation buffer A was 110 mM KCl, 2.24 mM MgCl_2_, 10 mM Tris HCl, 5 mM KH_2_PO_4_, 4 IU/ml creatine kinase, 50 mM creatine (pH 7.2 at 37°C). 5 mM succinate (plus 1 μM rotenone) was used as respiratory substrate. Mitochondrial state 3 respiration rate was achieved by adding 1 mM ATP in the presence of creatine kinase and creatine, which convert the ATP into ADP. The kinetics of the respiratory chain was determined as the dependence of the mitochondrial respiration rate on the membrane potential, when the latter was titrated with carboxyatractyloside (0.4–8 nmol/mg mitochondrial protein). The kinetics of the proton leak was measured as the rate of mitochondrial respiration in the presence of excess oligomycin 1 μg/mg protein (to prevent phosphorylation). This was determined over a range of different values of membrane potential by titrating with malonate (0.33–6 mM) – an inhibitor of the respiratory chain.

Isolated heart mitochondria were preincubated 1–2 min at room temperature in hypotonic conditions: 1 volume of mitochondrial suspension (at 20 mg/ml in isolation buffer) was added to 2 volumes of de-ionized water (so the final buffer contained 60 mM KCl, 6.7 mM Tris, and 0.3 mM EGTA, pH 7.3). Then 500 μM pyruvate plus 500 μM malate, 100 μM cGMP, 100 μM ATP and 40 IU/ml PKG Iα (bovine, recombinant; from Calbiochem), phosphatase inhibitor cocktail (1:100 dilution; from Sigma) were added and incubated 15 min at room temperature. Control mitochondria were preincubated in the same way only without PKG. Afterwards aliquots containing 0.2–0.5 mg mitochondrial protein were taken and added to 1 ml of the mitochondrial incubation buffer B (135 mM KCl, 30 mM Tris, 5 mM KH_2_PO_4_, 5 mM nitrilo-triacetic acid (NTA), 1.5 mM MgCl_2_, 1 mM pyruvate plus 1 mM malate, pH 7.2), and mitochondrial respiration, swelling and calcium accumulation were measured.

Mitochondrial respiration was measured in the incubation buffer B containing 135 mM KCl, 30 mM Tris, 5 mM KH_2_PO_4_, 5 mM NTA, 1.5 mM MgCl_2_, 1 mM pyruvate plus 1 mM malate, pH 7.2 as described above. State 3 respiration rate was initiated by adding 1 mM ADP. Mitochondrial swelling was assayed in buffer B by measuring a decrease in absorbance at 540 nm after addition of 250 μM CaCl_2_. After 15 min incubation mitochondria were centrifuged at 13000 rpm × 3 min in an Eppendorf centrifuge and supernatants were used for spectrophotometric measurements of cytochrome *c *as described in [19,20].

Mitochondrial calcium retention capacity was measured fluorimetrically using Calcium Green-5N (Molecular Probes, excitation at 506 nm, emission at 535 nm) which detects extramitochondrial Ca^2+^. The incubation buffer C contained 200 mM sucrose, 10 mM Tris-HCl, 1 mM KH_2_PO_4_, 10 μM EGTA, 0.3 mM pyruvate plus 0.3 mM malate, pH 7.4, 25°C, final volume 3 ml [21]. Calcium Green concentration in the medium was 100 nM. Calibration of the signal was achieved by the addition of known amounts of Ca^2+^. Experiments were started by the addition of 0.2 mg/ml mitochondria, then 10 μM CaCl_2 _pulses were added approximately every 3 min (after ~90% of added calcium was taken up by mitochondria) until opening of permeability transition pore occurred which was recorded as a large increase in fluorescence due to release of accumulated Ca^2+ ^from mitochondria.

For measurement of cytochrome *c*, mitochondria were solubilized with 1% Triton X-100 (w/v). Sodium hydrosulphite-reduced minus hydrogen-peroxide-oxidized absorption spectra difference was recorded with a Hitachi-557 spectrophotometer. Cytochrome *c+c*_1 _content was estimated by using the absorption difference at the wavelength pair 550/535 nm and ε = 14.5 mM^-1^cm^-1 ^as described in [20]. The cytochrome *c *content in cytosolic fractions was detected using Quantikinine M rat/mouse Immunoassay ELISA kit (R&D Systems). Cytosolic fraction proteins were dissolved in 0.5% Triton X-100 and further procedures were performed according to the manufacturer's protocol.

Lactate dehydrogenase (LDH) activity in the coronary effluent was measured spectrophotometrically [22] by monitoring the rate of decrease in NADH (at 340 nm) as pyruvate is converted to lactate. The LDH activity was measured by addition of effluent to 0.1 M Tris-HCl buffer (pH 7.5) containing 0.1 mM NADH and 1 mM Na-pyruvate. A unit of LDH was defined as the amount of enzyme necessary to catalyze oxidation of 1 μmol NADH per min (IU).

The activity of caspases was measured as described in [15]. Cytosolic fractions were prepared from heart homogenates in sucrose-based medium (250 mM sucrose, 5 mM HEPES, 2 mM EGTA, 1 mg/ml albumin) by differential centrifugation: 5 min × 750 g, 10 min × 6800 g, and 30 min × 10000 g. Sucrose-based medium rather than KCl-based buffer was used to avoid possible inhibition of caspase activity by high potassium salt concentrations. 1 mg/ml of total cytosolic protein was incubated for 30 min in buffer containing 10% sucrose, 50 mM HEPES, 1 mM MgCl_2_, 1 mM ATP (pH 7.4, 37°C) and 0.1 mM z-DEVD-p-nitroanilide (Alexis), a caspase-3 substrate. The hydrolysis of caspase substrate was followed spectrophotometrically at 405 nm and was calibrated with p-nitroanilide. DEVD-cleaving activity was completely suppressed by 0.02 mM DEVD-CHO (Alexis), a reversible inhibitor of caspase-3.

Data are expressed as means ± S.E. of at least 3 separate experiments. Statistical comparison between experimental groups was performed by ANOVA followed by Tukey or LSD tests. A value of p < 0.05 was considered statistically significant.

## Results

### DETA/NO protects mitochondrial functions from ischemic damage

We investigated whether a 'pure' NO donor DETA/NO can protect heart mitochondria from ischemia-induced mitochondrial damage and by what mechanism. We have previously shown that heart ischemia results in an inhibition of the mitochondrial respiratory chain (largely due to cytochrome *c *release) and a stimulation of mitochondrial proton permeability (largely due to increased free fatty acids) [18]. So we tested here whether NO could prevent these changes, by perfusing rat hearts with 50 μM DETA/NO for 3 min prior to 30 min of stop-flow ischemia. Mitochondria were then rapidly isolated from these and untreated control hearts, and the activities of the respiratory chain and proton leak were determined by simultaneously measuring mitochondrial respiration and membrane potential. We found that 30 min ischemia caused a 39% decrease in the state 3 respiratory rate (369 ± 26 nmol O/min/mg protein in control and 224 ± 11 nmol O/min/mg protein in ischemic mitochondria, p < 0.001) and this was prevented when hearts were pre-perfused with DETA/NO prior to ischemia (respiratory rate in ischemic DETA/NO-treated mitochondria was 292 ± 27 nmol O/min/mg protein, p < 0.01 compared to ischemic mitochondria). Perfusion of the heart with DETA/NO alone had no effect on the respiration rates of mitochondria isolated from non-ischemic hearts (361 ± 15 and 327 ± 28 nmol O/min/mg protein in control and DETA/NO group) or on the mitochondrial membrane potential (148 ± 3 mV and 148 ± 2 mV in state 3 for mitochondria from control and DETA/NO-perfused hearts, respectively).

**Figure 1:**
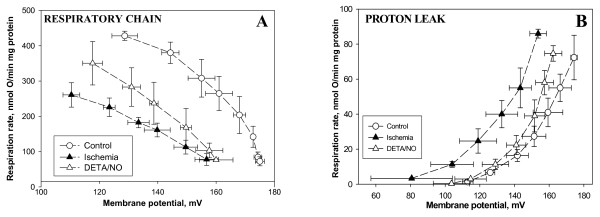
**Perfusion of hearts with DETA/NO partially prevents the ischemia-induced decrease in respiratory chain activity and increase in proton leak of subsequently isolated mitochondria**. (A): kinetics of the respiratory chain activity, and (B): kinetics of proton leak. Rat hearts were perfused for 3 min with 50 μM of DETA/NO, then ischemia was induced for 30 min. Isolated mitochondria were placed in a vessel with incubation buffer A (see Methods) and their oxygen consumption and membrane potential were measured simultaneously. 5 mM succinate (plus 1 μM rotenone) was used as respiratory substrate. Kinetics of the respiratory chain was determined as the dependence of the mitochondrial respiration rate on the membrane potential by titrating with carboxyatractyloside (0.4–8.0 nmol/mg mitochondrial protein). The kinetics of the proton leak was measured in the presence of excess oligomycin (1 μg/mg protein), mitochondrial respiration and membrane potential were titrated with malonate (0.33–6 mM). Means ± standard errors of 5 separate experiments are presented.

Fig. 1A shows the kinetics of the respiratory chain (with respiratory substrate succinate, in the presence of rotenone) of mitochondria isolated from control, ischemic and ischemic + DETA/NO-treated hearts. The kinetics of the respiratory chain were measured in isolated heart mitochondria as the dependence of the mitochondrial respiratory rate on membrane potential, when this was decreased by carboxyatractyloside (0.4–8 nmol/mg mitochondrial protein). The important point about these measured kinetics is that they are independent of the mitochondrial permeability or phosphorylation system, but rather reflect the overall activity of the respiratory chain and other components generating membrane potential in mitochondria [23]. As can be seen from the decreased respiration rates of ischemic mitochondria compared to control at any given membrane potential, ischemia caused a strong inhibition of the respiratory chain (Fig. 1A). However, after pre-perfusion of hearts with 50 μM DETA/NO, the respiratory chain of ischemia-damaged mitochondria had a higher activity compared to DETA/NO-untreated ischemic mitochondria (Fig 1A), i.e. NO partially prevented ischemic damage to the respiratory chain.

The kinetics of the proton leak were measured (Fig. 1B) as the dependence of the mitochondrial respiration rate in the presence of excess oligomycin (1 mg/mg protein used to inhibit phosphorylation) on membrane potential when this was decreased by malonate (0.33–6 mM). In these conditions, the respiration rate is proportional to the rate of proton leak, and these kinetics reflect the permeability of the inner membrane to protons [24]. As can be seen from Fig. 1B, at each particular value of membrane potential, the respiration rate of ischemic mitochondria was at least twice that of control mitochondria. This indicates that ischemia increases the mitochondrial proton leak, which we have previously attributed to an increase in free fatty acids [18]. However, pre-perfusion of the heart with DETA/NO largely prevented the ischemia-induced increase of proton leak (Fig 1B).

**Figure 2:**
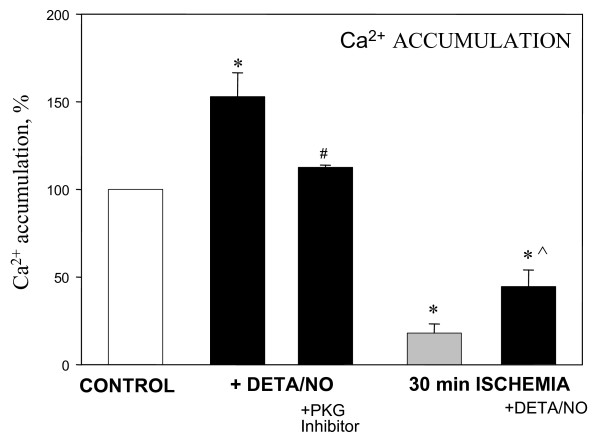
**Perfusion of hearts with DETA/NO increases the calcium-retention capacity of subsequently isolated mitochondria**. Rat hearts were perfused with Krebs-Henseleit solution only (control group) or with 50 μM DETA/NO for 3 min (DETA/NO group) or with 1 μM of PKG inhibitor KT 5823 for 5 min prior to perfusion with DETA/NO (DETA/NO + PKG inhibitor group). 30 min ischemia was induced as described in Methods. Isolated mitochondria (0.2 mg protein) were added to 3 ml incubation buffer C (200 mM sucrose, TrisHCl 10 mM, KH_2_PO_4 _1 mM, EGTA 10 μM, 0.3 mM pyruvate plus 0.3 mM malate, pH 7.4) and mitochondrial Ca^2+ ^accumulation was measured fluorimetrically using Calcium Green-5N (excitation at 506 nm, emission at 535 nm) as described in Methods. Ca^2+ ^retention capacity (amount required to be accumulated before large increase in fluorescence was observed due to opening of permeability transition pore) of control mitochondria was 124.6 ± 6.5 nmol/mg protein and was equated to 100%. * – statistically significant effect (p < 0.05, Tukey test) if compared to control, # – statistically significant effect of PKG inhibitor (p < 0.05, LSD test) if compared to DETA/NO group, ^ – statistically significant effect of DETA/NO if compared to ischemic group (p < 0.05, Tukey test). Means ± standard errors of 3–5 separate experiments are presented.

We additionally studied whether treatment of the heart with DETA/NO would increase the capacity of subsequently isolated mitochondria to retain calcium. This was assayed by measuring the extramitochondrial Ca^2+ ^concentration fluorimetrically with Calcium Green-5N. The addition of CaCl_2 _(2.5 μM) resulted in an immediate increase of extramitochondrial Ca^2+ ^concentration (measured as an increase in fluorescence), followed by Ca^2+ ^accumulation in the mitochondrial matrix (decrease of fluorescence), and subsequent additions of CaCl_2 _were repeated until the opening of the permeability transition pore occurred, which was recorded as a large, irreversible increase in fluorescence. We found that in mitochondria isolated from non-ischemic DETA/NO-perfused hearts 45% more calcium was necessary to cause permeability transition compared to control mitochondria (Fig. 2). But when hearts were perfused with both DETA/NO and the PKG inhibitor, the Ca^2+ ^retention capacity was significantly reduced (relative to mitochondria from hearts perfused with DETA/NO alone), and were similar to the untreated control level (Fig. 2). Mitochondria isolated from hearts after 30 min ischemia had a much lower capacity to accumulate Ca^2+ ^compared to control mitochondria, however, this capacity was significantly (by 2.5-fold) increased if the hearts were pre-perfused with DETA/NO prior to induction of 30 min ischemia. This indicates that NO treatment of heart increases the capacity of mitochondria to accumulate calcium, presumably by activating PKG which inhibits permeability transition.

Altogether, the data suggest that NO, in a PKG-dependent manner, protects mitochondrial function from ischemic damage mainly by preserving the intactness of the mitochondrial membranes: preventing the inner membrane from becoming leaky to protons, and outer membrane from becoming leaky for cytochrome *c*.

### DETA/NO prevents ischemia-induced cytochrome c release, caspase activation and necrosis via PKG

**Figure 3:**
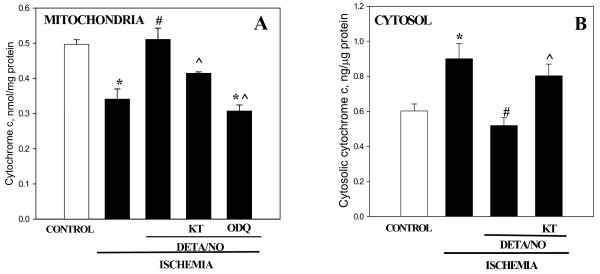
**Perfusion of hearts with DETA/NO prevents ischemia-induced release of cytochrome *c *from mitochondria**. (A) – mitochondrial cytochrome *c*, (B) – cytosolic cytochrome *c*. Content of cytochrome *c *in isolated mitochondria was measured spectrophotometrically from the characteristic spectra as described in Methods and was expressed as nmol/mg of mitochondrial protein. Cytosolic cytochrome *c *content was measured using ELISA kit and was expressed in ng/μg of cytosolic protein. * – Statistically significant effect of ischemia if compared to control (p < 0.01, Tukey test); # – statistically significant effect of DETA/NO if compared to ischemia (p < 0.01, Tukey test); ^ – statistically significant effect of KT 5823 (p < 0.05, LSD test) or ODQ (p < 0.01, Tukey test) if compared to ischemia + DETA/NO group. Means ± standard errors of 4–9 separate experiments are presented.

Next we investigated whether DETA/NO could prevent the ischemia-induced release of cytochrome *c *from mitochondria to cytosol and subsequent caspase activation in perfused rat hearts. After 30 min of heart ischemia, the mitochondrial cytochrome *c *content had decreased by 31% compared to the non-ischemic control (Fig. 3A). 3 min pre-perfusion with 50 μM DETA/NO completely protected mitochondria from cytochrome *c *loss: mitochondrial cytochrome *c *content in ischemic hearts treated with DETA/NO was not different from non-ischemic control levels. Cytochrome *a/a*_3 _remained unchanged in all treatments (data not shown) indicating that the mitochondrial inner membrane was not damaged and the changes in cytochrome *c *spectra were relatively specific for cytochrome *c *content. Concomitant with the decrease of mitochondrial cytochrome *c *during ischemia, there was an increase in cytosolic cytochrome *c*, and this was prevented when hearts were pre-perfused with DETA/NO (Fig 3B). Selective PKG inhibitor KT5823 at 1 μM concentration applied together with DETA/NO before ischemia significantly decreased the ability of DETA/NO to prevent cytochrome *c *loss from mitochondria and its accumulation in cytosol: mitochondrial cytochrome *c *level was significantly reduced and cytosolic level significantly increased in DETA/NO plus KT5823 perfused ischemic hearts compared to DETA/NO perfused hearts (Fig. 3A and 3B). Similarly, ODQ – a selective inhibitor of NO-sensitive guanylyl cyclase, abolished the protective effect of DETA/NO against ischemia-induced release of cytochrome *c *from mitochondria (Fig. 3A).

**Figure 4:**
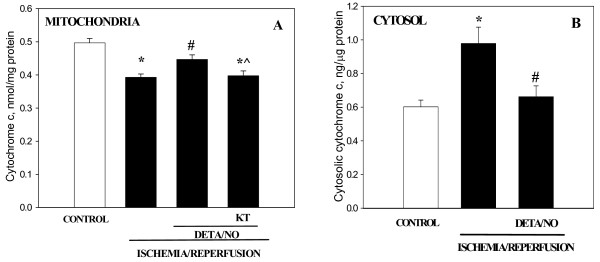
**Perfusion of hearts with DETA/NO protects heart mitochondria from the loss of cytochrome c after ischemia/reperfusion**. (A) – mitochondrial cytochrome *c*, (B) – cytosolic cytochrome *c*. Experimental conditions were the same as described in Fig. 3, except that rat hearts after perfusion with DETA/NO (± KT 5823) were subjected to 30 min stop-flow global ischemia followed by 30 min reperfusion. * – Statistically significant effect of ischemia/reperfusion (I/R) (p < 0.01, Tukey test) if compared to control; # – statistically significant effect of DETA/NO (p < 0.05, Tukey test) if compared to I/R. ^ – statistically significant effect of KT 5823 (p < 0.05, LSD test) if compared to I/R + DETA/NO group. Means ± standard errors of 4–9 separate experiments are presented.

Similar results were obtained when the heart was reperfused after ischemia (Fig. 4). The amount of mitochondrial cytochrome *c *after 30 min ischemia followed by 30 min reperfusion was by 21% lower compared to the control (Fig. 4A). Pre-treatment with DETA/NO partially blocked this ischemia/reperfusion-induced loss, and the protective effect of DETA/NO was completely eliminated by PKG inhibitor KT5823 (Fig. 4A). The cytochrome *c *content of cytosolic extracts from ischemia/reperfused hearts increased by 63% compared to control, while in hearts pre-treated with DETA/NO there was no significant increase in cytosolic cytochrome *c *compared to the non-ischemic control level (Fig. 4B). These data indicate that NO, acting via PKG, prevents the cytochrome *c *release from mitochondria to cytosol induced by both ischemia and ischemia plus reperfusion.

It has been shown that PKG may open mitochondrial K^+^_ATP _channels (mtK_ATP_) [14,25], which may play a role in cardioprotection. Therefore we tested whether the effect of DETA/NO on mitochondrial cytochrome *c *release might be related to opening of mtK_ATP _channels. 5-hydroxydecanoate (5-HD) blocks the mtK_ATP _channel, and perfusion of heart with 5-HD has been shown to block NO-induced protection against ischemia/reperfusion-induced necrosis [26]. However, we found that 15 min pre-perfusion with 100 μM 5-HD did not alter DETA/NO-induced protection against ischemia-induced cytochrome *c *release: mitochondrial cytochrome *c *content in nmol/mg mitochondrial protein was 0.43 ± 0.03 in control, 0.30 ± 0.02 in 30 min ischemic, 0.37 ± 0.01 in DETA/NO-pretreated ischemic, and 0.37 ± 0.01 in DETA/NO- plus 5-HD-pretreated ischemic mitochondria (n = 4) suggesting that the protective effect of DETA/NO is not mediated through mtK_ATP _channels. Perfusion with DETA/NO, KT5823 or 5-HD alone had no effect on mitochondrial cytochrome *c *content.

**Figure 5:**
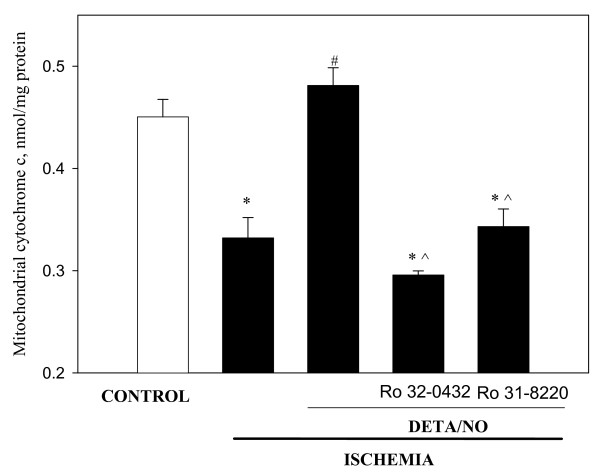
**Perfusion of the hearts with PKC inhibitors abolish the protective effect of DETA/NO against ischemia-induced loss of cytochrome *c *from mitochondria**. Hearts were perfused 15 min with 0.5 μM Ro 32-0432 and 1 μM Ro 31-8220 followed by 3 min perfusion with 50 μM DETA/NO. Then hearts were subjected to 30 min stop-flow ischemia. Mitochondrial content of cytochrome *c *was measured as described in Methods. * – Statistically significant effect of ischemia if compared to control (p < 0.01, Tukey test); # – statistically significant effect of DETA/NO if compared to ischemia (p < 0.01, Tukey test); ^ – statistically significant effect of PKC inhibitors if compared to DETA/NO group (p < 0.05, Tukey test). Means ± standard errors of 4 separate experiments are presented.

Recently it has been reported that protein kinase C_ε _(PKC_ε_) may be a downstream target of PKG in mitochondria inhibiting mitochondrial permeability transition pore [25,27]. To test whether PKC is involved in DETA/NO-induced protection of heart mitochondria against ischemia-induced loss of cytochrome *c *we pre-perfused the hearts with DETA/NO in the presence of selective inhibitors of PKC – Ro 32-8220 and Ro 31-0432, and then subjected the hearts to 30 min ischemia. As summarised in Fig. 5, both inhibitors of PKC practically eliminated the protective effect of DETA/NO: the levels of cytochrome *c *in mitochondria isolated from ischemic hearts treated with DETA/NO plus PKC inhibitors were as low as the levels in non-treated ischemic mitochondria and substantially lower than in ischemic mitochondria treated with DETA/NO only (Fig. 5). This suggests that PKC is involved in a DETA/NO-activated cascade of events leading to inhibition of cytochrome *c *release from mitochondria during ischemia.

**Figure 6:**
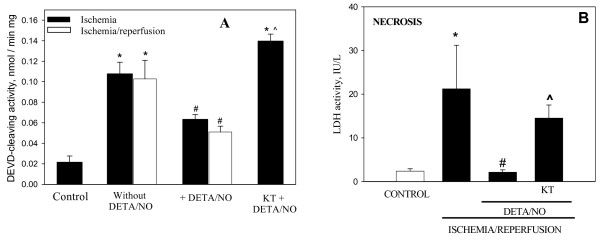
**Perfusion of the hearts with DETA/NO blocks caspase activation and necrosis induced by ischemia and ischemia/reperfusion**. (A) – caspase activity, (B) – lactate dehydrogenase activity in perfusate. Rat hearts were perfused for 3 min with 50 μM DETA/NO, then hearts were subjected to 30 min ischemia only or to 30 min ischemia plus 30 min reperfusion. Where indicated (KT), hearts were perfused with 1 μM KT5823. Coronary effluents during reperfusion were collected for determination of LDH activity. Cytosolic fractions were separated by differential centrifugation and were used for determination of caspase activity spectrophotometrically using z-DEVD-*p*-nitroanilide, a caspase-3-substrate. * – Statistically significant effect of ischemia (I) or I/R (p < 0.01, Tukey test) if compared to control; # – statistically significant effect of DETA/NO (p < 0.05, Tukey test) if compared to I or I/R respectively. ^ – statistically significant effect of KT5823 (p < 0.05, LSD test) if compared to I/R + DETA/NO group. Means ± standard errors of 4–9 separate experiments are presented.

Release of cytochrome *c *from mitochondria to cytosol can cause caspase activation, and this has previously been shown to occur in perfused heart after ischemia [15]. In this work we tested whether pre-ischemic perfusion of hearts with DETA/NO would have any effect on caspase activation in ischemic or ischemic and reperfused hearts. As can be seen in Fig. 6A, 30 min ischemia induced a 5-fold increase in caspase activity compared to the (non-ischemic) control, and in the presence of DETA/NO, the ischemia-induced activation of caspases was significantly reduced (by 54%). The protective effect of DETA/NO was eliminated when the hearts were pre-perfused with PKG inhibitor KT2358: the level of ischemia-induced caspase activation in this group (KT2358 and DETA/NO treatment before ischemia) was as high as in ischemic group (Fig. 6A). In the ischemia/reperfused hearts, the caspase activation and its blockage by DETA/NO were very similar to ischemia alone (Fig 6A). These data indicate that NO protects the heart from ischemia-induced apoptosis via PKG activation.

We also tested the effect of DETA/NO on the level of ischemia/reperfusion-induced necrosis by monitoring lactate dehydrogenase (LDH) activity in the perfusate. After 30 min ischemia plus 30 min reperfusion, LDH activity in the perfusate markedly increased (Fig. 6B), and pre-perfusion with DETA/NO reduced the LDH activity in the perfusate back to the control level, indicating that cells were completely rescued from ischemia/reperfusion-induced necrosis. KT5823 applied together with DETA/NO restored necrosis back to a level similar to that induced by ischemia/reperfusion without DETA/NO, indicating that PKG mediates the cardioprotective action of DETA/NO.

### The effects of PKG on the functional activity of isolated mitochondria

**Figure 7:**
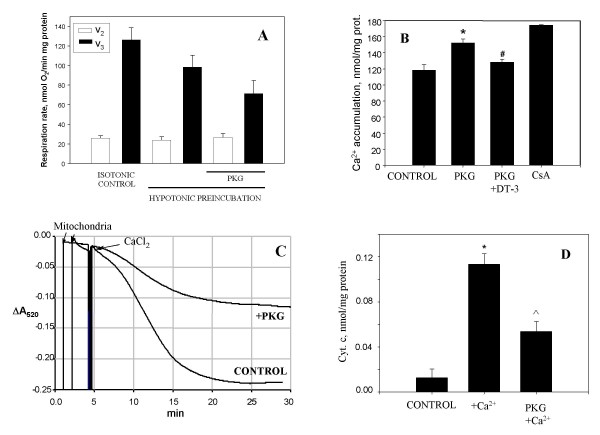
**The effects of purified PKG on the functional activities of isolated heart mitochondria**. (A) – mitochondrial state 2 and state 3 respiration rates, (B) – mitochondrial Ca^2+ ^accumulation, (C) – mitochondrial swelling, (D) – release of cytochrome *c *from mitochondria. Isolated heart mitochondria were preincubated in hypotonic conditions with 40 U/ml PKG Iα as described in Methods. Then mitochondria (0.2–0.5 mg) were added to 1 ml incubation buffer B (plus 1 mM pyruvate and 1 mM malate as respiration substrates) and mitochondrial respiration and swelling were measured. Mitochondrial swelling was assayed by measuring a decrease in absorbance at 540 nm after addition of 250 μM CaCl_2_. After 15 min incubation mitochondria were removed by centrifugation, the supernatants were used for spectrophotometric measurements of released of cytochrome *c*. Mitochondrial calcium accumulation was measured in buffer C fluorimetrically using 100 nM Calcium Green-5N (excitation at 506 nm, emission at 531 nm) as described in Methods. DT-3 – protein kinase G Iα inhibitor (from Calbiochem). * – statistically significant effect of PKG (or Ca^2+^) (p < 0.05, Tukey test) if compared to control, # – statistically significant effect of PKG inhibitor DT-3 (p < 0.05, Tukey test) if compared to mitochondria + PKG group, ^ – statistically significant effect of PKG (p < 0.05, Tukey test) if compared to mitochondria plus Ca^2+^. Means ± standard errors of 7 separate experiments are presented.

To investigate in more detail which mitochondrial processes can be influenced by activated PKG we studied the direct effects of PKG on the functions of isolated mitochondria. Isolated heart mitochondria were preincubated in hypotonic conditions for 1–2 min and then 15 min with/without added PKG (plus cGMP). This mildly hypotonic preincubation had no significant effect on mitochondrial respiration compared to isotonic conditions, but these hypotonic conditions were needed to reveal a stable effect of PKG on mitochondrial functions. PKG added to isolated mitochondria slightly reduced respiration rate in state 3 but had no effect in state 2 (Fig. 7A). However, this inhibition did not reduce the capacity of these mitochondria to accumulate added calcium. This was assayed by measuring the extramitochondrial Ca^2+ ^concentration fluorimetrically with Calcium Green-5N as described above (in Fig. 2). After hypotonic pre-incubation with/without PKG mitochondria were transferred to the buffer C and pulses of CaCl_2 _were added until the opening of the permeability transition pore occurred, which was recorded as a large, irreversible increase in fluorescence. The control mitochondria were able to accumulate 111 ± 5 nmol/mg protein of calcium, however the PKG-treated mitochondria had a higher calcium retention capacity: 152 ± 3 nmol/mg protein (Fig. 7B). Inhibition of PKG with DT-3 returned the calcium retention capacity back to almost the control level (Fig. 7B). As expected, the presence of cyclosporine A (CsA) to inhibit permeability transition resulted in a higher calcium retention capacity (175 ± 1 nmol/mg protein).

Next we investigated whether PKG could block mitochondrial swelling triggered by high concentrations of Ca^2+^, which is known to be mediated by permeability transition. As can be seen in Fig. 7C, high-amplitude swelling of isolated mitochondria was induced by 250 μM CaCl_2_, while pre-treatment with PKG significantly blocked this swelling.

The swelling of mitochondria was accompanied by the release of cytochrome *c *from mitochondria to incubation medium (Fig. 7D). In these experiments, the isolated mitochondria were preincubated for 15 min in conditions as described in Fig. 7C, then mitochondria were removed by centrifugation and supernatants were used for spectrophotometric measurements of cytochrome *c*. There was no detectable loss of cytochrome *c *from mitochondria when they were incubated without Ca^2+^. After incubation of mitochondria with 250 μM CaCl_2_, there was a substantial increase in the cytochrome *c *level in supernatants compared to calcium-free control. This calcium-induced cytochrome *c *release was substantially reduced by the addition of PKG (Fig 7D). These results suggest that PKG increases the resistance of isolated mitochondria to the deleterious effects of high calcium: the swelling and release of cytochrome *c *due to opening of the permeability transition pore were substantially reduced by PKG.

## Discussion

We have previously shown that 30 min of heart ischemia causes cytochrome *c *release from the mitochondria to the cytosol, and this causes an inhibition of respiratory chain function, which can be reversed simply by adding back exogenous cytochrome c [15]. Here we show that a pure NO donor (DETA/NO) can reduce these changes, probably via a PKG-mediated blockage of mitochondrial permeability transition and subsequent cytochrome *c *release. Ischemia-induced cytochrome *c *release is important because: (a) it stimulates caspase activation in the cytoplasm, and (b) it may contribute to reperfusion-induced necrosis or contractile dysfunction, either because it prevents ATP production or because it stimulates reactive oxygen species production from the respiratory chain. We find here, consistent with [14,28], that DETA/NO does indeed protect against reperfusion-induced necrosis via a PKG-dependent mechanism. We have previously shown that addition of cytochrome *c *to heart cytosol (in the presence of dATP) is sufficient to trigger caspase activation, and blocking cytochrome *c *release blocks ischemia-induced caspase activation and nuclear apoptosis [15,29]. We show here that pre-perfusion of hearts with DETA/NO blocks the ischemia-induced cytochrome *c *release and caspase activation. It is still not entirely clear how ischemia-induced caspase activation contributes to heart pathology, but caspase inhibition has been shown to protect the heart in animal models of ischemia/reperfusion [16,17].

Ischemia substantially increased the mitochondrial proton permeability and pre-perfusion with DETA/NO prevented this. We have previously attributed this increased proton permeability to the measured ischemia-induced increase in free fatty acids within the mitochondria, as the permeability increase is reversed simply by incubating the mitochondria with albumin [18]. It is possible that NO blocks the ischemia-induced increase in free fatty acids, as it has been shown that cGMP-stimulated PKG can phosphorylate and inhibit cytosolic phospholipase A2 [30], which is a likely source of the ischemia-induced increase in free fatty acids. And the free fatty acids may both increase proton permeability and stimulate permeability transition. Alternatively, it may be that mitochondrial permeability transition contributes to the proton permeability directly or indirectly.

Incubation of isolated heart mitochondria with activated PKG blocked calcium-induced mitochondrial permeability transition and cytochrome *c *release. We used a mildly hypotonic medium to obtain these results, because although we could also see protection by PKG in an isotonic medium with some preparations of mitochondria, the results varied from day to day, whereas there was consistent protection by PKG using a hypotonic medium. This medium was only mildly hypotonic and had little effect on the state 3 or state 2 respiratory rate, indicating that inner and outer mitochondrial membranes were not ruptured. However, we can not rule out that there was some mild or transient permeabilisation of the outer membrane allowing PKG to access intermembrane target proteins that mediated the protection. Alternatively the hypotonic treatment may have been required to form contact sites between the inner and outer membrane that allow changes at the outer membrane to be conveyed to the inner membrane [31]. In this hypotonic medium, activated PKG blocked calcium-induced permeability transition, as measured by swelling and calcium retention. This suggests that PKG phosphorylated some mitochondrial component that controls permeability transition.

It has previously been shown that activated PKG can block permeability transition in isolated brain [32], heart [25] and liver mitochondria [33], although the mechanism of the blockage remains unclear. In heart a complex pathway has been invoked including PKG activating PKC_ε1_, activating mtK_ATP_, increasing matrix pH, increasing H_2_O_2_, activating PKC_ε2_, and inhibiting permeability transition [25]. However, we found that a mtK_ATP _inhibitor did not prevent NO inhibiting the ischemia-induced cytochrome *c *release. It is possible that NO via PKG reduces the sensitivity of mtK_ATP _to 5-HD [14], such that mtK_ATP _is not inhibited by 100 μM 5-HD in the presence of NO. Alternatively, mtK_ATP _is not involved in NO protection against apoptosis (rather than necrosis). This protection may involve NO activation of sGC to produce cGMP, which activates PKG, which somehow activates mitochondrial PKC to inhibit permeability transition, which normally mediates ischemia-induced cytochrome *c *release. This would be consistent with a recent report by Costa et al. [27] showing that exogenous H_2_O_2 _or NO inhibit MPTP opening independently of mtK_ATP _activity via activation of mitochondrial PKC_ε2_.

## Conclusion

Overall our results indicate that NO can acutely block ischemia-induced caspase activation, mitochondrial damage and cell death in the heart via PKG, and this may be mediated by PKG inhibition of mitochondrial permeability transition and related loss of cytochrome *c *from mitochondria.

## Competing interests

The authors declare that they have no competing interests.

## Authors' contributions

VB planed the design of the study, performed experiments on the effects of PKG on isolated mitochondria, measured nitric oxide, analysed the data, and wrote the manuscript. RM performed measurements of mitochondrial and cytosolic cytochromes, necrosis, and performed the statistical analysis. OA carried out heart perfusions and measurements of mitochondrial respiration and calcium retention capacity. AJ performed the kinetic analysis of mitochondrial respiratory chain and proton leak. JB measured activities of caspases. GCB conceived the study, participated in its design and coordination and helped to draft the manuscript. All authors read and approved the final manuscript.
